# Effectiveness of a Parent Empowerment Program for Parents of Children with Autism: A Randomized Controlled Trial

**DOI:** 10.1111/cch.70148

**Published:** 2025-08-02

**Authors:** Damla Şahin Büyük, Dilek Özmen

**Affiliations:** ^1^ Dept. of Public Health Nursing Faculty of Health Sciences, Manisa Celal Bayar University Manisa Türkiye

**Keywords:** autism, care burden, empowerment, parent training, parental self‐efficacy, parental stress

## Abstract

**Background:**

Parents of children with autism often face significant stress, low self‐efficacy, and caregiver burden in meeting their children's complex needs. This study evaluated the effectiveness of a parent empowerment program combining parental training and motivational interviewing to support caregivers of children with autism in Türkiye.

**Methods:**

A total of 69 parents (interventio*n* = 34, control = 35) participated in this unblinded, two‐group randomized controlled study, which was conducted between September 2020 and May 2022. A parent empowerment program, including four parental training sessions and two motivational interview sessions, was applied to the parents in the intervention group. The Parental Self‐Efficacy Scale, Zarit Care Burden Scale, Perceived Stress Scale and Family Empowerment Scale were used to evaluate the effectiveness of the empowerment program. Standard practice was performed for the control group.

**Results:**

Parents in the intervention group showed significantly greater improvements than those in the control group in self‐efficacy (*t* = 5.340, *p* < 0.001), perceived stress (*t* = −4.636, *p* < 0.001) and family empowerment (*t* = 2.745, *p* = 0.008). No significant difference was observed between the groups in caregiver burden (*p* = 0.086).

**Conclusion:**

This study reveals that using a parent empowerment program that includes motivational interviews along with training interventions is effective in empowering parents to manage their children's care, reducing stress, and supporting them to acquire effective parenting skills by increasing self‐efficacy. Future research should explore designs that assess the independent and combined effects of motivational interviews and parent training programmes in randomised controlled trials.

The study was registered at ClinicalTrials.gov (https://clinicaltrials.gov/) under the registration number NCT06629974 on October 8, 2024.

## Summary

- Combining parent training with motivational interviewing can effectively increase self‐efficacy and reduce stress among parents of children with autism, which is a critical factor in sustaining long‐term caregiving.
- Empowering parents through structured psychoeducational support improves their ability to manage daily care challenges, contributing to more consistent and effective child‐centred care.
- Motivational interviewing, as a client‐centred and flexible method, is particularly beneficial for tailoring support to the diverse and evolving needs of families with children on the autism spectrum.
- Integrating multidimensional psychosocial interventions into existing autism rehabilitation services may enhance family functioning, promote caregiver resilience, and support better developmental outcomes for children.

## 1 Introduction

Autism spectrum disorder (ASD), commonly known as autism, is a typically lifelong neuropsychiatric condition characterised by deficits in social interaction and communication skills, along with restricted, repetitive behaviours and interests (American Psychiatric Association 2013). It is estimated that about one in every 100 children worldwide has autism (Zeidan et al. 2022). However, this represents an average figure, and the prevalence of autism can vary significantly between studies. According to recent data from the US Centres for Disease Control and Prevention (CDC), the prevalence of autism in the United States is 1 in 44 (Maenner et al. 2021). Many low‐ and middle‐income countries lack data on the prevalence of autism (Zeidan et al. 2022). Although Türkiye lacks comprehensive nationwide epidemiological data on autism, available reports and expert assessments suggest that the prevalence has been increasing in line with global trends (Yaylaci and Guller 2025).

The abilities and needs of individuals with autism can vary and evolve. While some people with autism can live independently, others may have severe limitations and require lifelong care and support (Lord et al. 2020). The uncertainty surrounding the causes of ASD, coupled with its variable severity and duration, makes it difficult for families to adapt. Research indicates that parents of children with autism encounter more challenges than parents of those with other disabilities (Hayes and Watson 2013; May and Williams 2024; Padden and James 2017; Yorke et al. 2018). Some parents with a child with autism may sometimes lose confidence in managing their child's care and feel inadequate. Meeting the needs of a child with autism, coping with problematic behaviours, keeping a child who is unaware of dangers under constant supervision and simultaneously addressing the demands of other family members is a very stressful and exhausting process. During this time, parents may experience stress, intense panic and guilt, making it difficult to fulfil all the expected roles (Marcinechová et al. 2024; Strauss et al. 2024). Parents of children with autism, who must juggle many roles intensively, often experience high levels of care burden (Bozkurt et al. 2019; Patel et al. 2022). Parents with high self‐efficacy manage stress and its negative effects better, experiencing less care burden (Kishimoto et al. 2023; Kurzrok et al. 2021; Li et al. 2022; Strauss et al. 2024). Therefore, research on parental self‐efficacy emphasizes the importance of multidimensional psychosocial support, professional support interventions and parental adaptation to increase parents' resilience (Albanese et al. 2019; Hayes and Watson 2013; Iadarola et al. 2018). Family support services for parents of children with autism in Türkiye are currently limited and unevenly distributed. With much of the support offered by non‐governmental or private actors, disparities in access are common, which may further intensify caregiving challenges, especially among families with lower socioeconomic status. Such disparities highlight the potential value of structured and accessible family support programs (Rfat et al. 2023).

Parents' lack of knowledge on various issues related to their children with autism is one of the most significant factors contributing to their tension and anxiety. Providing appropriate educational support mechanisms for parents on how to care for their children with autism can be a great source of strength for families (Lichtle et al. 2020). Various studies have shown that educational interventions with different content for parents of children with autism have positive effects on reducing parents' care burden (Johnson et al. 2019), stress (Akhani et al. 2021; Rohacek et al. 2023), self‐efficacy (Albanese et al. 2019; Iadarola et al. 2018) and family functioning (Gentile et al. 2022; Minjarez et al. 2013). Given the diverse challenges faced by parents of children with autism, it is essential to enhance their motivation regarding their care and parenting roles by providing both emotional and educational support in care management (Johnson et al. 2023; Leung et al. 2022). In this context, motivational interviewing (MI) can be used as a method to empower parents. MI, a tool for helping individuals understand their problems and take action for change, is effective in improving parental self‐efficacy and coping with psychiatric issues such as stress, anxiety and depression (Almansour et al. 2023; Larson et al. 2023). Each individual with autism is unique, which means the challenges faced by parents with children diagnosed with autism can vary greatly. The flexibility of MI, which does not prescribe standard goals and allows for client‐centred work, makes it particularly advantageous for parents of children on the highly variable autism spectrum (Almansour et al. 2023). An intervention that combines parent training with MI is crucial for enhancing child care, improving family functioning and maintaining family integrity. Such an intervention can also help parents mitigate the negative effects of stress, reduce the burden of care and increase their sense of competence in managing their children's care (Rohacek et al. 2023). While some studies evaluate the effectiveness of parent training interventions in autism (Gentile et al. 2022; Lichtle et al. 2020), research on the combined use of MI and training is limited (Rohacek et al. 2023). This study aims to evaluate the effectiveness of an empowerment program that includes both parent training and MI for parents of children with autism, supporting their competence in the care of their children.

## 2 Methods

### 2.1 Research Design and Sampling

**FIGURE 1:**
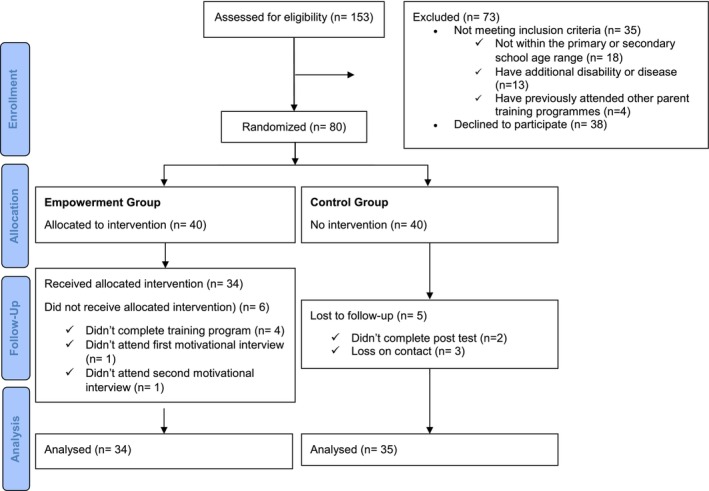
Flowchart of participant (adapted from http://www.consort‐statement.org/).

This unblinded, two‐parallel‐group randomised controlled interventional study was conducted between September 2020 and May 2022. The a priori sample size was calculated using G*Power Version 3.1.9.2, based on a similar study in the literature (Hemdi and Daley 2017). The effect size for repeated measures was determined to be f: 0.282. With an alpha level of 0.05 (type I error probability) and a power of 0.80 (1‐β error probability), the minimum sample size required was calculated to be 64 participants (32 in the intervention group and 32 in the control group). Data were collected from public schools and special education rehabilitation centres officially affiliated with the Ministry of National Education in the study region. To account for potential attrition during the study, 80 participants were initially included, 40 in the intervention group and 40 in the control group. Due to various reasons (in the control group, two participants did not complete the post‐test and three could not be reached; in the intervention group, four participants did not complete the training sessions and two did not attend all motivational interview sessions), there were some losses in the sample during the study, and it was completed with a total of 69 participants: 34 in the intervention group and 35 in the control group (Figure 1).

After the data collection process, a Post Hoc G‐Power analysis was performed to evaluate the study's power with the final sample size. The analysis revealed that the study had 99% power, based on a 95% confidence interval, a significance level of *p* = 0.05, and the calculated mean score and standard deviation of the Parental Self‐Efficacy Scale.

### 2.2 Inclusion and Exclusion Criteria

Inclusion criteria were having a child diagnosed with ASD who was in primary or secondary school (typically ages 6 to 14), being literate and volunteering to participate in the study. This age range was selected as it covers the years during which parents face similar challenges in managing their child's care, communication skills and behavioural issues (Toper and Özkan 2021).

Exclusion criteria included having a child with a concomitant disability or disease (e.g., epilepsy, attention deficit and hyperactivity disorder, other physical or mental disabilities), previous participation in an education programme with similar content, or parents who did not participate in at least two‐thirds of the empowerment training programme or did not attend all motivational interview sessions. These participants were excluded from the study, and their data were not included in the analysis.

### 2.3 Randomization

Potential participants were recruited through rehabilitation centres and schools in the city centre. Initially, 153 parents were assessed for eligibility, and 73 were excluded for various reasons (Figure 1). The remaining 80 participants were randomly assigned to either the intervention or control group using simple randomisation with a table of random numbers. To minimise selection bias, the randomisation was carried out by an independent academic staff member from a different department who was not involved in the research design, data collection, analysis, or interpretation. The homogeneity of the groups was assessed using a chi‐square test, which showed no significant differences in key demographic variables (*p* > 0.05).

### 2.4 Data Collection Tools

#### 2.4.1 Introductory Information Form

The Introductory Information Form comprised 20 items developed by the researchers to obtain demographic and background information relevant to the study. The questions addressed variables such as the parent's age, gender, marital status, education level, occupation, income level, number of children, as well as the age, gender, age at diagnosis and autism severity of the child. The form was developed based on a review of existing literature and similar studies in the field (Bozkurt et al. 2019; Hayes and Watson 2013; Hemdi and Daley 2017; Iadarola et al. 2018; Iida et al. 2018).

#### 2.4.2 The Parental Self‐efficacy Scale

The scale was developed by Guimond et al. (2005) to measure the self‐efficacy of parents of children with disabilities regarding parenting skills (Guimond et al. 2005). The Turkish adaptation of the scale was first made by Diken (2007) and then by Cavkaytar et al. (2014) (Cavkaytar et al. 2014; Diken 2007). The Parental Self‐Efficacy Scale is a seven‐point Likert‐type rating scale consisting of 17 items. The lowest and highest values that can be taken from the scale are 17 and 119. Higher scores indicate higher self‐efficacy perceptions. The Cronbach Alpha internal consistency coefficient value of the scale was calculated as 0.95 (Cavkaytar et al. 2014). The Cronbach's Alpha internal consistency coefficient value of the scale in this study is 0.96.

#### 2.4.3 The Zarit Care Burden Scale

The Zarit Care Burden Scale was developed by Zarit et al. in 1980 and adapted to Turkish by Inci and Erdem (2006). The scale is a 22‐item, five‐point Likert‐type scale used to evaluate the stress experienced by caregivers of individuals in need of care. A minimum score of 0 and a maximum score of 88 can be obtained from the scale. The higher the score obtained from the scale, the higher the care burden felt. The Cronbach's Alpha internal consistency coefficient value of the scale was found to be 0.75 in the original study (Inci and Erdem 2006; Zarit et al. 1980) and 0.89 in this study.

#### 2.4.4 The Perceived Stress Scale (PSS‐14)

The Perceived Stress Scale was developed by Cohen et al. (1983) and adapted into Turkish by Eskin et al. This 14‐item, five‐point Likert‐type scale has two sub‐dimensions: inadequate self‐efficacy perception and stress/discomfort perception. Higher scores indicate higher levels of perceived stress. The Cronbach's alpha internal consistency coefficient for the entire scale was 0.90, with the sub‐dimensions scoring 0.86 and 0.83, respectively (Cohen et al. 1983; Eskin et al. 2013). In this study, the Cronbach's alpha internal consistency coefficient was found to be 0.75 for the overall scale and 0.73 and 0.75 for the sub‐dimensions, respectively.

#### 2.4.5 The Family Empowerment Scale

The Family Empowerment Scale, developed by Singh et al. (1995) and adapted into Turkish by Karakul et al. (2018), consists of 34 questions and three sub‐dimensions, rated on a four‐point Likert scale. Higher scores indicate stronger family empowerment. The Cronbach's alpha internal consistency coefficient for the entire scale was 0.89. The sub‐dimensions had the following Cronbach's alpha values: 0.79 for the Family subdimension, 0.83 for the Service system subdimension and 0.79 for the Community/Political sub‐dimension (Karakul et al. 2018; Singh et al. 1995). In this study, Cronbach's alpha coefficient was 0.79 for the overall scale, 0.83 for the family dimension, 0.89 for the services sub‐dimension and 0.85 for the community/political sub‐dimension.

### 2.5 Data Collection

#### 2.5.1 Intervention Group

Participants in this group were administered the pre‐intervention introductory information form, the Parental Self‐Efficacy Scale, the Zarit Care Burden Scale, the Perceived Stress Scale and the Family Empowerment Scale. Then, a group empowerment training programme consisting of four sessions in total was applied to the participants. Two individual motivational interview sessions were conducted with each participant in the intervention group 10 days after the empowerment training programme. One month after the motivational interview sessions were completed, post‐test data were collected.

#### 2.5.2 Control Group

Standard practice was performed for the control group. The data collection tools applied to the intervention group in the pre‐test and post‐test were also applied to the control group. Following the completion of the study, the control group was granted access to the educational materials used in the intervention group's training programme.

### 2.6 Scope of the Empowerment Program

The empowerment program, developed by the researchers, consisted of two parts: the empowerment training program for parents and motivational interviews.

#### 2.6.1 Empowerment Training Program

The empowerment training program was prepared to enable parents to carry out the care process more effectively in areas where parents have difficulties caring for their children with autism. Within the scope of the empowerment training program, parents were trained on the importance of play and communication, problems and management in the field of nutrition, sleep problems and management, safety and protection from accidents and problems and management in the field of self‐care.

To determine the training content, qualitative interviews were initially conducted with 17 parents of children diagnosed with autism. Alongside these interviews, the literature was reviewed to create the training material. Once the content was developed based on the qualitative interviews and literature review, it was evaluated for suitability by a panel of seven experts. This panel included two special education teachers working with autistic groups, two psychological counsellors in special education, one academician specialising in autism, one parent of a child with autism and one head of an autism‐related non‐governmental organisation. The content validity index (CVI) was calculated as 0.95 using the Davis technique (Davis 1992), confirming the appropriateness of the training materials. The training sessions were delivered according to this predefined content, ensuring consistency in implementation. To maintain adherence to the planned topics, session checklists were used throughout the training. This approach helped ensure that all key components were covered systematically and uniformly across sessions.

Considering that group interaction processes could positively influence the learning experience for parents, the training was conducted as face‐to‐face group sessions. These sessions were organized into four 45‐min segments, with a 15‐min break between each session. The training was delivered using a combination of interactive methods, including structured presentations, guided discussions and question‐and‐answer sessions to enhance engagement and knowledge retention.

#### 2.6.2 Motivational Interview Sessions

Ten days after the empowerment training programme, two face‐to‐face motivational interviews were conducted with each participant in the intervention group. Each participant had a one‐week break between the first and second motivational interviews. Each motivational interview session lasted approximately 25–30 min.

To ensure fidelity and competence in MI, the researcher conducting the sessions completed a 16‐h training programme covering both theoretical and practical aspects of MI techniques. This training was designed to ensure adherence to established MI principles and techniques, thereby enhancing the effectiveness of the sessions.

### 2.7 Data Analysis

The SPSS 25.0 package program was used to evaluate the data. Numbers, percentages and descriptive statistics were used to describe demographic characteristics, and the chi‐square test was used to evaluate the homogeneity of the groups. The normality of the data distribution was assessed using skewness and kurtosis values. Since both coefficients were within the widely accepted ±2 threshold, the data were considered to meet the normality assumption, and parametric tests were deemed appropriate for analysis (George and Mallery 2010). *t*‐Test in independent groups was used for the comparison of scale mean scores between the intervention and control groups since the data were normally distributed, and *t*‐test in dependent groups was used for the comparison of mean scores between baseline and post‐intervention measurements. The results were evaluated to be within the 95% reliability interval and *p* < 0.05 statistically significant level.

### 2.8 Ethical Considerations

Ethical approval was received for this study from the Manisa Celal Bayar University Health Sciences Ethics Committee (No = 20.278.486). Official permission was received from the Provincial Directorate of National Education for the relevant schools and special education rehabilitation centres. Written and verbal consent was also obtained from the parents involved in the study.

## 3 Results

Although female gender was not a selection criterion in this study, all of the parents who voluntarily participated in the study were women. The mean age of the participants was 38.82 ± 5.46 (min‐max = 28.00–50.00). Most of the participants had male children with autism, and most of the children had moderate autism. There was no statistically significant difference (*p* > 0.05) between the intervention and control groups in terms of all descriptive characteristics evaluated for both participants and their children (Table 1 and Table 2).

**TABLE 1:** Descriptive characteristics of participants in the intervention and control group.

Characteristics	Intervention (*n* = 34)		Control (*n* = 35)		Total		x^2^/*p*
	*n*	%	*n*	%	*n*	%	
Gender							
Female	34	100	35	100	69	100	
Age *38.82 ± 5.46 (min‐max = 28.00–50.00)							
≤39	16	47.1	19	54.3	35	50.7	0.360/0.548
≥ 39	18	52.9	16	45.7	34	49.3	
Marital status							
Married	30	88.2	29	82.9	59	85.5	0.367/0.477**
Single	4	11.8	6	17.1	10	14.5	
Family type							
Nuclear	28	82.4	23	65.7	51	73.9	2.476/0.171
Other	6	17.6	12	34.3	18	26.1	
Income level							
Income less than expenses	16	47.1	13	37.1	29	42.0	0.696/0.404
Income equal to expenses	18	52.9	22	62.9	40	58.0	

* Mean ± standard deviation, x^2^ = chi square test.

** Fisher's exact chi‐square test.

**TABLE 2:** Descriptive characteristics of the child diagnosed with ASD.

Characteristics	Intervention (*n* = 34)		Control (*n* = 35)		Total		x^2^/*p*
	*n*	%	*n*	%	*n*	%	
Gender of the child							
Girl	11	32.4	12	34.3	23	33.3	0.290/0.865
Boy	23	67.6	23	65.7	46	66.7	
Age of the child *9.30 ± 2.23(Min = 6.00, Max: 14.00)							0.153/0.696
≤9 years old	21	61.8	20	57.1	41	59.4	
≥ 9 years old	13	38.2	15	42.9	28	40.6	
Child's age of diagnosis							
2–3 years old	21	61.8	20	57.1	41	59.4	0.153/0.696
4–5 years old	13	38.2	15	42.9	28	40.6	
Severity of autism (according to CARS‐2)							
Mild	10	29.4	11	31.4	21	30.4	0.369/0.832
Moderate	12	35.3	14	40.0	26	37.7	
Severe	12	35.3	10	28.6	22	31.9	
Maternal education level							
Primary education	12	35.3	15	42.9	27	39.2	0.888/0.683
High school	12	35.3	13	37.1	25	36.2	
University	10	29.4	7	20.0	17	24.6	
Paternal education level							
Primary education	6	17.6	8	22.9	14	20.3	0.438/0.847
High school	21	61.8	19	54.3	40	58.0	
University	7	20.6	8	22.9	15	21.7	

* Mean ± standard deviation, x^2^ = chi square test.

**FIGURE 2:**
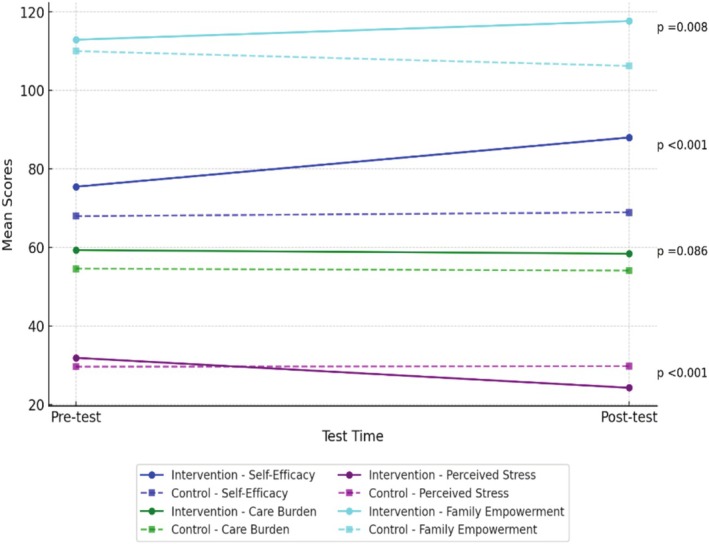
Comparison of pre‐test and post‐test scores between groups.

Table 3 and Figure 2 present the comparisons of the Parental Self‐Efficacy Scale, Zarit Care Burden Scale, Perceived Stress Scale and Family Empowerment Scale scores between the intervention and control groups (Table 3, Figure 2).

**TABLE 3:** Comparison of the parental self‐efficacy scale, the zarit care burden scale, the perceived stress scale and the family empowerment scale scores in the intervention and control groups.

Scale		Intervention (*n* = 34)	Control (*n* = 35)
		Test statistics
Effect size value		
	Mean ± SD	Mean ± SD
The Parental Self‐Efficacy Scale	Pre‐test	75.44 ± 12.69	67.94 ± 18.85	t = 1.942 *p* = 0.057	Cohen's d = 1.283
	Post‐test	87.97 ± 11.87	68.91 ± 17.33	t = 5.340 ***p* < 0.001**	
Test statistics		*t = −7.195/***p* < 0.001**	*t = −0.676/*p* = 0.504		
The Zarit Care Burden Scale	Pre‐test	59.29 ± 9.04	54.57 ± 11.16	t = 1.933 *p* = 0.058	Cohen's d = 0.419
	Post‐test	58.38 ± 9.19	54.08 ± 11.20	t = 1.743 *p* = 0.086	
Test statistics		*t = 1.073/*p* = 0.295	*t = 0.427/*p* = 0.672		
The Perceived Stress Scale	Pre‐test	31.85 ± 5.93	29.60 ± 5.02	t = 1.700 *p* = 0.094	Cohen's d = 1.114
	Post‐test	24.23 ± 3.90	29.74 ± 5.80	t = −4.636 ***p* < 0.001**	
Test statistics		*t = 5.584/***p* < 0.001**	*t = −0.137/*p* = 0.892		
The Family Empowerment Scale	Pre‐test	112.88 ± 12.95	109.97 ± 22.76	t = 0.650 *p* = 0.518	Cohen's d = 0.663
	Post‐test	117.61 ± 11.32	106.20 ± 21.53	t = 2.745 ***p* = 0.008**	
Test statistics		*t = −4.319/***p* < 0.001**	*t = 1.839/*p* = 0.075		

*Note:* t = t‐test in Independent Groups. Bold values indicate statistically significant differences (*p* < 0.05).

* t = t‐test in Dependent Groups.

At post‐test, the intervention group reported significantly higher scores in parental self‐efficacy (*t* = 5.340, *p* < 0.001, Cohen'SD = 1.283) and family empowerment (t = 2.745, *p* = 0.008, Cohen'SD = 0.663) compared to the control group. Additionally, perceived stress levels were significantly lower in the intervention group than in the control group (*t* = −4.636, *p* < 0.001, Cohen'SD = 1.114) (Table 3).

In contrast, no significant between‐group difference was found for caregiver burden (*t* = 1.743, *p* = 0.086, Cohen'SD = 0.419), although the effect size suggests a small to moderate trend favouring the intervention (Table 3).

In Cohen's d effect size classification, an effect size of 0.2 is considered small, 0.5 medium and 0.8 large effect (Rosenthal et al. 1994). In this study, the effect of the intervention (Cohen's d) ranged between 0.419 and 1.283 (Table 3).

## 4 Discussion

Parents of children with autism often face significant challenges in addressing their child's health, educational and care needs. Limited knowledge and insufficient support can intensify these difficulties, leading to heightened stress and disrupted family dynamics. Multidimensional support is therefore essential. This study examines the outcomes of a comprehensive empowerment programme that includes both training and motivational interviews for parents of children with autism.

Parental self‐efficacy refers to parents' beliefs in their ability to perform parenting tasks successfully (Gross and Rocissano 1988). Parents with high self‐efficacy are more likely to consistently engage in positive parenting practices and manage challenging parenting situations effectively, but individuals with low self‐efficacy are more inclined to give up (Russell and Ingersoll 2021). The empowerment program intervention implemented in this study was found to be effective in improving parental self‐efficacy. Consistent with the literature, this finding supports our initial predictions (Iadarola et al. 2018; Shiri et al. 2020) and can be considered a result of the fact that the implemented intervention makes parents more effective in managing the issues that parents have difficulties in caring for their children, believing that they have the necessary ability in this regard, increasing their motivation, and determination and exhibiting the necessary behaviours (Albanese et al. 2019).

It is important for the development of their children that parents with children with autism have lower stress levels or can manage their stress effectively (Crowell et al. 2019). The parent empowerment programme implemented in this study was found to be an effective intervention in reducing parental stress. Rohacek et al. (2023) conducted a randomised controlled study to evaluate the effect of a behaviour‐based parent training programme (BPT) on the problem behaviours of children with autism and parental stress. They applied BPT to the intervention group and a psychoeducation and supportive therapy programme (PST), including MI, to the control group. Their findings showed no significant difference between the intervention and control groups in terms of the variables addressed for both parents and children. Rohacek et al. attributed this to the motivational interviews within the PST creating a significant change in the control group, thus eliminating the difference between the two groups. In other words, using the PST intervention, which is also a strong activity, in the control group against the BPT intervention caused no significant change between the groups. The findings of Rohacek et al.'s study overlap with the findings of this study in demonstrating the effectiveness of parent training and motivational interviews in reducing parental stress (Rohacek et al. 2023). Several other research studies corroborate the findings of this study (Akhani et al. 2021; Iida et al. 2018; Shiri et al. 2020; Singh et al. 2021). In contrast, Johnson et al. (2023) reported different findings in their randomised controlled trial where they evaluated a telehealth education programme for parents of children with autism. They did not observe a significant reduction in parental stress. One possible reason for this discrepancy could be that telehealth education programmes might be less effective than face‐to‐face education programmes in alleviating parental stress. However, Johnson et al. focused only on addressing sleep disorders in children with autism, whereas parents often face multiple behavioural challenges that contribute to their stress. This narrow focus on sleep disorders may have limited the overall impact on reducing parental stress (Johnson et al. 2023). Also, the parent empowerment programme implemented in this study included both parent training and motivational interviews, which likely contributed to its significant results across various outcomes. Moreover, parent empowerment programmes may also have indirect positive effects on children's development and overall family dynamics. By strengthening parents' self‐efficacy, emotional regulation and problem‐solving skills, such programmes can help create a more supportive and structured home environment, positively influencing children's behavioural and emotional outcomes (Banach et al. 2010). Similarly, MI has consistently been shown to enhance caregiving behaviours and parental engagement by fostering empathy, autonomy and confidence, which can indirectly benefit child well‐being (Larson et al. 2023).

One of the key findings of this study is the program's effectiveness in strengthening family dynamics, which underscores the broader benefits of comprehensive support for parents of children with autism (Banach et al. 2010; Gentile et al. 2022; Minjarez et al. 2013). During the group training, parents had valuable opportunities to learn from each other's experiences in caring for their children and coping with family challenges (Boshoff et al. 2021). Additionally, the motivational interviews conducted as part of the empowerment program allowed parents to reflect on their personal experiences and current situations, which they may not have had the chance to explore amidst their busy lives. The role of motivational interviews in helping parents understand their challenges and take proactive steps toward change likely contributed to the positive impact observed in family empowerment outcomes (Rogers et al. 2019).

The unexpected result that the empowerment program did not significantly alleviate parental care burdens, contrary to initial expectations, highlights the intricate challenges faced by parents of children with autism. Care burden entails a spectrum of difficulties spanning physical, psychological, economic, emotional and social dimensions (Schene et al. 1998). Many factors affect the burden of care; therefore, addressing these multifaceted challenges demands holistic interventions that encompass educational support, healthcare services, psychological counselling and economic and social assistance (Peer and Hillman 2014). However, the current intervention was limited to psycho‐education and MI, without broader social or economic support components that are often pivotal in easing caregiver burden. This limited scope may explain the lack of statistically significant change. Nonetheless, the moderate effect size (Cohen's d = 0.42), despite the absence of statistical significance, suggests a meaningful trend that might have emerged more clearly with a larger sample or extended follow‐up.

Effective reduction of care burden often requires collaborative efforts across various sectors and specialised expertise, along with governmental support, to implement comprehensive interventions. Therefore, this result may have been influenced by the fact that the empowerment programme potentially addressed only a subset of the broader support needs. Furthermore, the persistent caregiving role typically assumed by mothers, particularly in many cultural contexts, may have contributed to the enduring care burden despite the intervention's efforts. Despite not having selection criteria, all participants in this study were women, reflecting prevalent cultural norms where women bear primary responsibility for household tasks and childcare, which can explain this finding. There are studies with similar (Johnson et al. 2019) and contrasting results (Mai and Chaimongkol 2022) regarding our study's findings. These discrepancies may be attributed to variations in intervention programme characteristics, such as scope, duration and sample size strength.

### 4.1 Limitations

This study has several limitations to consider. First, following the empowerment training programme in the intervention group, no interim data collection or evaluation was conducted before the motivational interviews, which prevented the independent assessment of each intervention's specific effects. Second, although female gender was not a selection criterion, all voluntarily participating parents were women. Third, only two motivational interview sessions were conducted per participant. Additionally, while the study included parents of children aged 6 to 14 years to address shared caregiving challenges, developmental differences within this range may have influenced parental experiences and intervention outcomes. Future research should consider age‐specific interventions tailored to developmental needs.

The study also assessed only short‐term outcomes, and the sustainability of effects over time remains uncertain. The cultural context of the sample where caregiving roles are traditionally expected to be fulfilled by mothers may have influenced parental perceptions of care burden, stress and self‐efficacy. This socio‐cultural factor should be taken into account when interpreting the findings.

All outcomes were based on parent self‐reports, which may have introduced social desirability and recall bias. Additionally, the single‐site recruitment from an urban centre limits the generalisability to rural or culturally diverse settings, highlighting the need for multi‐site research. Data were also collected during the COVID‐19 pandemic, a time of elevated stress and disrupted services; thus, replication under post‐pandemic conditions is recommended. Lastly, the absence of a certified MITI 4.2.1 coder within the research team precluded formal fidelity assessment of MI sessions.

## 5 Conclusion

The findings of this study provide evidence that the parent empowerment programme supports parents of children with autism in various ways. This educational intervention reveals that the use of motivational interviews along with educational interventions is effective in empowering parents to manage their children's care, enhancing self‐efficacy and developing effective parenting skills. By integrating MI and structured parent education, this study offers a novel and practical intervention model that contributes to the growing body of literature on family‐centred autism care. It highlights the potential of combined approaches to strengthen caregiver outcomes in real‐life settings. Future researchers are encouraged to conduct randomised controlled trials using a design that enables separate and combined evaluations of MI and parent education programmes. Additionally, removing limits on the number of motivational interview sessions and making the parent education programme continuously available to parents upon request outside of scheduled sessions may enhance the robustness of findings and improve outcomes.

## Author Contributions

**Damla Şahin Büyük:** conceptualisation, methodology, formal analysis, funding acquisition, writing – original draft, writing – review and editing. **Dilek Özmen:** conceptualisation, methodology, funding acquisition, writing – review and editing.

## Ethics Statement

Ethical approval was received for this study from the Manisa Celal Bayar University Health Sciences Ethics Committee (No = 20.278.486).

## Conflicts of Interest

The authors declare no conflicts of interest.

## Data Availability

The data that support the findings of this study are available on request from the corresponding author. The data are not publicly available due to privacy or ethical restrictions.
